# Supplementary material for: Low-Value Clinical Practices: Knowledge and Beliefs of Spanish Surgeons and Anesthetists
Source: Int J Environ Res Public Health. 2020 May 19;17(10):3556. doi: 10.3390/ijerph17103556 (PMC7277874; doi:10.3390/ijerph17103556)
Supplement: Supplementary file 1 [file ijerph-17-03556-s001.pdf]

Supplemental File for Review – Survey Instrument

The overuse of the health system compromises the patient safety in an unwarranted way and involves a loss of opportunity cost.

Through this research, funded by Instituto de Salud Carlos III through the project PI16/00971, we aim to analyze this issue and to develop recommendations for improve the patient safety.

Several professionals and research societies of different Spanish regions (Andalucía, Aragón, Castilla La Mancha, Comunidad Valenciana, Madrid, Murcia, Navarra y País Vasco) collaborates in this study.

We request your collaboration asking you to fill this questionnaire, according to your professional experience. The survey aims to explore the frequency and causes of low value surgical practices.

Please, be aware that the questionnaire must be done once per participant. Estimated time for complete the questionnaire is around 7 minutes.

With the fulfillment and sending of this questionnaire, you show your consent for participate in this study.

Thank you for your collaboration.

1. According to your experience ¿What kind of unwarranted procedures are the most requested by the patients? (mark all options you consider properly)

☐ [1.1] Electrocardiogram

☐ [1.2] Computed axial tomography

☐ [1.3] Magnetic resonance

☐ [1.4] Other radiological studies

☐ [1.5] Antibiotic therapy

☐ [1.6] Non-steroidal anti-inflammatories

☐ [1.7] Prostate Specific Antigen

☐ [1.8] Routine check-up analytics

☐ [1.9] Cytology

☐ [1.10] Bone densitometry

☐ [1.11] Referral to other specialists

☐ [1.12] Other procedures (free text space)

2. According to your experience. What is the patient profile which usually requests unwarranted health procedures?

2.1 Gender

- ☐ Female
- ☐ Male
- ☐ DK/DA

2.2 Age

- ☐ 18 to 30 years old
- ☐ 31 to 50 years old
- ☐ 51 to 65 years old
- ☐ ≥ 65 years old
- ☐ DK/DA

2.3 Patient's Health Situation

- ☐ Multi-pathological patient'
- ☐ Low prevalence disease
- ☐ Undiagnosed disease

☐ Patient consulting on the Internet

☐ DK/DA

☐ Other procedures (free text space)______________

3. Please, mark how often you are in the following situations:

	**Never**	**Monthly**	**Almost every week**	**Almost every day**	**Every day**
[3.1] Patients request unwarranted medical tests or procedures	☐	☐	☐	☐	☐
[3.2] You indicate unwarranted medical tests or procedures due to patient pressure	☐	☐	☐	☐	☐
[3.3] You persuade the patient that the unwarranted medical procedures he requests are not appropriate	☐	☐	☐	☐	☐
[3.4] You receive patient's negative or aggressive reaction after a justified refusal	☐	☐	☐	☐	☐

4. Please, mark the main causes that make you to indicate unwarranted health procedures (mark all options you consider properly)

☐ [4.1] To get more clinical control and patient safety.

☐ [4.2] Due to the habit of performing this procedure in your setting[4.3] Fear of being sued.

☐ [4.4] To get patient satisfaction.

☐ [4.5] To respect patient's expectations

☐ [4.6] Lack of time in the consultation.

☐ [4.7] For not knowing how to explain to the patient that it is unnecessary

☐ [4.8] To avoid a claim or complaint.

☐ [4.9] To conduct an epidemiological or clinical study.

☐ [4.10] Recommendations in obsolete guidelines.

☐ [4.11] Persistent patient pressure.

5. According to your experience, ¿What is the effectiveness level of the following arguments to persuade patient about the inadequacy of medical procedures?

	**None**	**Lack**	**Moderate**	**High**	**Very High**
[5.1] Clinical reasons based on knowledge.	☐	☐	☐	☐	☐
[5.2] Patient's own safety.	☐	☐	☐	☐	☐
[5.3] The obtaining of the same result by other tests or previous procedures.	☐	☐	☐	☐	☐
[5.4] Saving of time and costs that have a positive effect on other patients.	☐	☐	☐	☐	☐
[5.5] Saving of discomfort for the patient.	☐	☐	☐	☐	☐

6. According to your opinion, mark the degree of responsibility regarding overuse of the following collectives (0 means "Lowest responsibility " and 10 "Highest responsibility"):

6.1. Top management of health systems

Lowest responsibility	0	1	2	3	4	5	6	7	8	9	10	Highest responsibility
	☐	☐	☐	☐	☐	☐	☐	☐	☐	☐	☐	

6.2. Directors of healthcare setting

Lowest responsibility	0	1	2	3	4	5	6	7	8	9	10	Highest responsibility
	☐	☐	☐	☐	☐	☐	☐	☐	☐	☐	☐	

6.3. Medical coordinators / Heads of department

Lowest responsibility	0	1	2	3	4	5	6	7	8	9	10	Highest responsibility
	☐	☐	☐	☐	☐	☐	☐	☐	☐	☐	☐	

6.4. Medical Doctors

Lowest responsibility	0	1	2	3	4	5	6	7	8	9	10	Highest responsibility
	☐	☐	☐	☐	☐	☐	☐	☐	☐	☐	☐	

6.5. Nursing/other health professions

Lowest responsibility	0	1	2	3	4	5	6	7	8	9	10	Highest responsibility
	☐	☐	☐	☐	☐	☐	☐	☐	☐	☐	☐	

6.6. Patient's Associations

Lowest responsibility	0	1	2	3	4	5	6	7	8	9	10	Highest responsibility
	☐	☐	☐	☐	☐	☐	☐	☐	☐	☐	☐	

6.7. Mass media (Press, radio, television)

Lowest responsibility	0	1	2	3	4	5	6	7	8	9	10	Highest responsibility
	☐	☐	☐	☐	☐	☐	☐	☐	☐	☐	☐	

6.8. Managers of health sites on the Internet

Lowest responsibility	0	1	2	3	4	5	6	7	8	9	10	Highest responsibility
	☐	☐	☐	☐	☐	☐	☐	☐	☐	☐	☐	

7. According to your opinion, mark the association between the indication of unwarranted health procedures and defensive medicine.

Lowest association	0	1	2	3	4	5	6	7	8	9	10	Highest association
	☐	☐	☐	☐	☐	☐	☐	☐	☐	☐	☐	

8. Please, mark true or false.

	True	False
[8.1] The early detection of urinary tract infection must be mandatory in all patients with urinary catheter, with weekly infection screening cultures.	☐	☐
[8.2] Hair removal only must be done if it is compromised the surgical field visibility. If it is necessary, it must be done with electric shavers or chemical hair removal, never with shaving blades.	☐	☐
[8.3] The patient's consent prior any intervention or invasive procedure can be obtained in an auto-administrated way.	☐	☐
[8.4] The Prostate Specific Antigen, for the prostate cancer screening, must be indicated in every male patient older than 55 years old.	☐	☐
[8.5] For a whole preoperative assessment, it always must be indicated: chest radiography, biochemical analysis, complete blood count, and coagulation test.	☐	☐
[8.6] Peri-operative antibiotic prophylaxis must be indicated at least 1 hour before any intervention, with a remaining administration between 24 – 48 hours.	☐	☐
[8.7] All hospitalized patients must receive proton pomp inhibitor for gastroduodenal ulcer prophylaxis	☐	☐

9. According to your opinion, mark the degree of usefulness of an educational campaign to reduce the requirement of patients for unwarranted medical tests and procedures (0 means "Not agree at all" and 10 "Fully agree"):

Not agree at all	0	1	2	3	4	5	6	7	8	9	10	Fully agree
	☐	☐	☐	☐	☐	☐	☐	☐	☐	☐	☐	

10. Do you know the "Commitment to the Quality of Scientific Societies" (ICC) initiative, launched by the Spanish Ministry of Health, Social Services and Equality, collecting most relevant unwarranted clinical practices of each medical fields ("do not do practices")?

☐ Yes

☐ No

11. Professional Experience

☐ < 1 year

☐ 1 to 5 years

☐ 6 to 15 years

☐ 16 to 29 years

☐ ≥ 30

11. Gender

☐ Female

☐ Male
